# Network Preservation Analysis Reveals Dysregulated Synaptic Modules and Regulatory Hubs Shared Between Alzheimer’s Disease and Temporal Lobe Epilepsy

**DOI:** 10.3389/fgene.2022.821343

**Published:** 2022-03-02

**Authors:** Anna Harutyunyan, Nigel C. Jones, Patrick Kwan, Alison Anderson

**Affiliations:** ^1^ Department of Medicine, Royal Melbourne Hospital, University of Melbourne, Parkville, VIC, Australia; ^2^ Department of Neuroscience, Central Clinical School, Monash University, Melbourne, VIC, Australia; ^3^ Department of Neurology, The Alfred Hospital, Melbourne, VIC, Australia

**Keywords:** Alzheimer’s disease, EphA4, gene networks, network preservation analysis, SCN3B, temporal lobe epilepsy, synaptic signalling, WGCNA

## Abstract

**Background:** There is increased prevalence of epilepsy in patients with Alzheimer’s disease (AD). Although shared pathological and clinical features have been identified, the underlying pathophysiology and cause-effect relationships are poorly understood. We aimed to identify commonly dysregulated groups of genes between these two disorders.

**Methods:** Using publicly available transcriptomic data from hippocampal tissue of patients with temporal lobe epilepsy (TLE), late onset AD and non-AD controls, we constructed gene coexpression networks representing all three states. We then employed network preservation statistics to compare the density and connectivity-based preservation of functional gene modules between TLE, AD and controls and used the difference in significance scores as a surrogate quantifier of module preservation.

**Results:** The majority (>90%) of functional gene modules were highly preserved between all coexpression networks, however several modules identified in the TLE network showed various degrees of preservation in the AD network compared to that of control. Of note, two synaptic signalling-associated modules and two metabolic modules showed substantial gain of preservation, while myelination and immune system-associated modules showed significant loss of preservation. The genes *SCN3B* and *EPHA4* were identified as central regulatory hubs of the highly preserved synaptic signalling-associated module. *GABRB3* and *SCN2A* were identified as central regulatory hubs of a smaller neurogenesis-associated module, which was enriched for multiple epileptic activity and seizure-related human phenotype ontologies.

**Conclusion:** We conclude that these hubs and their downstream signalling pathways are common modulators of synaptic activity in the setting of AD and TLE, and may play a critical role in epileptogenesis in AD.

## Introduction

In the past 50 years, it has become apparent that there is increased prevalence of epileptic seizures in patients with AD compared to the general population (Hauser et al., 1986; McAreavey et al., 1992; Volicer et al., 1995; Hesdorffer et al., 1996; Vossel et al., 2013), but only recently have there been well-designed studies attempting to understand this link (Miranda and Brucki, 2014; Vossel et al., 2017). A 2006 study reported that patients with AD have ∼10-fold increased risk of developing seizures, with early onset or familial AD patients having as high as 87-fold higher risk (Amatniek et al., 2006). These patients show more severe cognitive impairment (McAreavey et al., 1992) and rapid disease progression (Volicer et al., 1995). Likewise, individuals with epilepsy show higher risk of developing dementia when compared to the general population (Forsgren et al., 1996; Pugh et al., 2009). In AD patients, epileptiform activity is commonly detected in temporal brain regions (Vossel et al., 2016), and temporal lobe epilepsy (TLE) is generally recognized as the subtype of epilepsy that has the most overlap in its pathophysiology with AD (Scharfman, 2012). In addition to electrical abnormalities and cognitive impairment, AD and TLE share pathological features such as amyloid deposition (Mackenzie and Miller, 1994), tau pathology (Thom et al., 2011; Tai et al., 2016) and hippocampal sclerosis (Davidson et al., 2011). Additionally, several transgenic animal models of AD exhibit spontaneous seizures and have increased susceptibility to epilepsy (Palop et al., 2007; Westmark et al., 2008; Ziyatdinova et al., 2011; Chan et al., 2015; Ziyatdinova et al., 2016; Reyes-Marin and Nuñez, 2017), suggesting the pathological hallmarks of AD may directly cause seizures.

Diseases as dysfunctional states are associated with altered gene expression, which can be detected by transcriptional analysis of the mRNA in a given tissue. However, genes do not operate in isolation, but rather interact cooperatively within and across biological pathways. Thus, it is insufficient to identify one differentially expressed group of genes in order to thoroughly characterize a disease. A more comprehensive understanding of the associated pathology requires capturing changes in biological pathways and their interaction. Viewing disease as the result of an elaborate interplay of cellular pathways - much like a network -accounts for the intricacy and complexity of human biology, as it assumes that perturbations in a single node of this network have the potential to affect the entire community or module it belongs to. This systems or network approach is proving to be powerful in biomarker discovery (Tran et al., 2011; Clarke et al., 2013; Huan et al., 2015; Sun et al., 2017) due to its multiple advantages over the traditional linear association model approach, which fails to fully account for the complex web of interactions of gene products and key regulators.

Weighted gene coexpression network analysis (WGCNA) is a widely used systems biology methodology that investigates the correlation between genes based on their expression level across all samples in the dataset (Zhang and Horvath, 2005). The genes (nodes) in the network are connected by an edge if the two genes have similar expression pattern, i.e., their expressions rise and fall together (correlated) or when one rises the other falls (anticorrelated) (Zhang and Horvath, 2005). The WGCNA method has valuable advantages over knowledge-based networks such as protein-protein interaction (PPI) networks as it is not biased in favour of the known protein-protein interactions of the member nodes. The resultant network is constructed solely based on the pairwise expression correlation pattern of all genes. These networks are then hierarchically clustered into highly connected groups of genes called modules, and further examined through functional enrichment analysis. It has been shown previously that modularity is a conserved property of biological systems and modules of genes, proteins or metabolites have functional significance, i.e. the cellular functions are carried out by these highly connected modules of genes/proteins (Oldham et al., 2006; Oldham et al., 2008). These functional modules tend to be extremely heterogeneous, wherein the majority of the nodes have relatively few connections (edges) with other nodes, thus rendering them less relevant in the overall function of the module, while a few “hub” nodes are highly connected and therefore are considered important regulators of the given module (Ravasz et al., 2002).

Once a healthy state and a dysfunctional state are defined in a gene coexpression network graph, its architecture becomes a comparable and quantifiable attribute that is representative of the system. The topology of these networks can then be investigated and compared in order to identify important gene regulators and capture the differential connectivity and preservation of modules, which in turn reflect the overlap in biological pathways implicated in the conditions the networks are associated with.

Recently, it has been proposed that disease phenotypes that were previously thought of as distinct entities may share common pathological mechanisms and have strong molecular relationships (Barabási et al., 2011). Given the correlated incidence and shared clinical symptoms between TLE and AD, we hypothesized that the common pathological features might be a result of a strong molecular relationship between the two diseases in the form of a shared set of perturbed cellular pathways and dysregulated gene modules. Since the electrophysiological and morphological symptoms common to TLE and AD impact the hippocampus in the setting of both diseases, we set out to compare the transcriptome of hippocampal tissue affected by TLE and AD. To achieve this, we employed the framework of WGCNA (Zhang and Horvath, 2005) to construct signature gene networks representing TLE and AD, and then employed network preservation statistics methods (Langfelder et al., 2011) to compare and contrast the signature gene networks by measuring the preservation of TLE modules in the AD coexpression network. Additionally, since functional gene modules have been shown to be preserved even across different species (Oldham et al., 2006; Miller et al., 2010), we also examined the preservation of TLE modules in a non-demented control (NDC) network to facilitate distinction of homeostatic (common to all networks) and pathology-specific (characteristic to disease state) features.

## Materials and Methods

### Data Pre-Processing, Normalization and Covariate Adjustment

**FIGURE 1:**
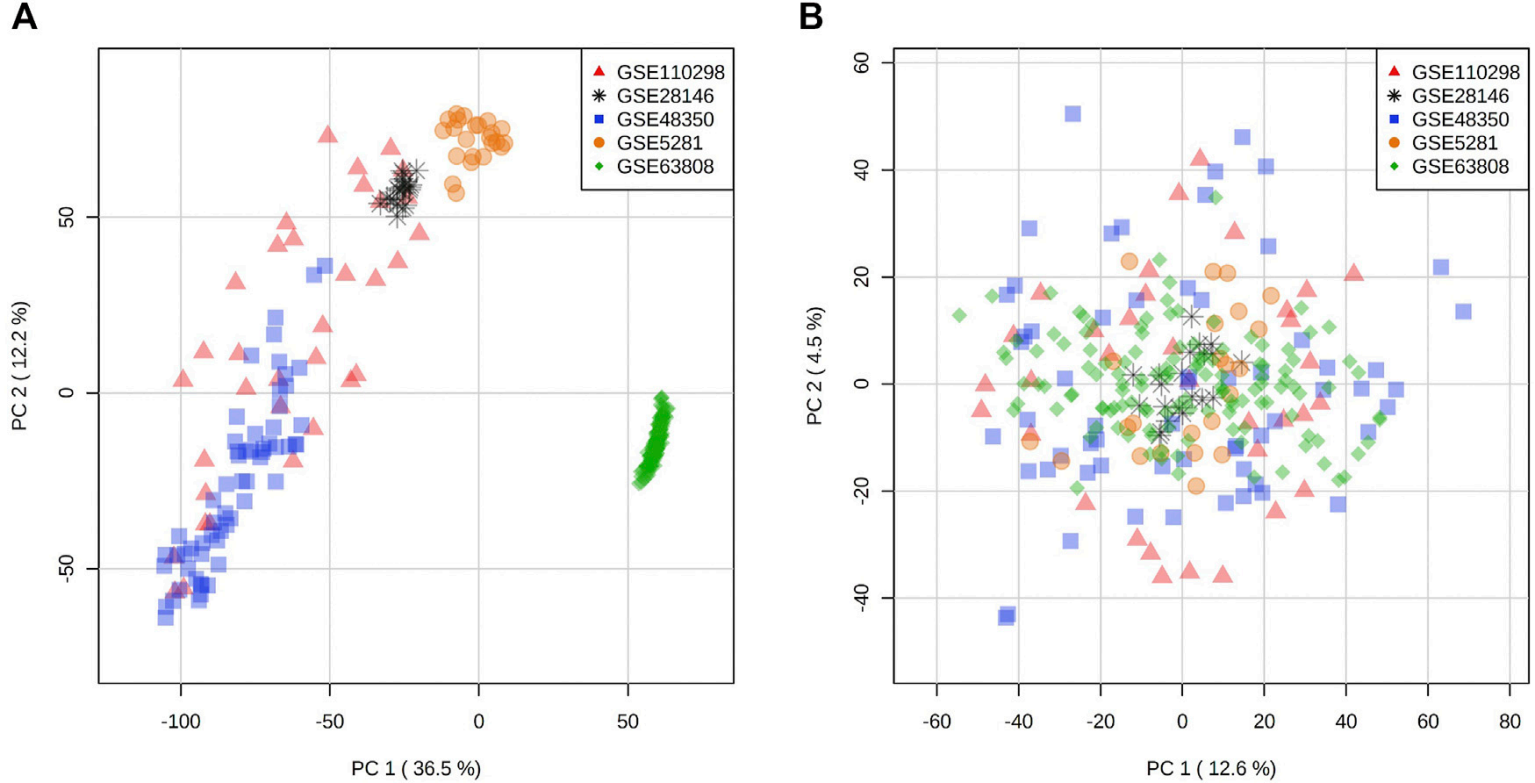
Principal component analysis of expression distribution of all datasets **(A)** before batch effect adjustment and **(B)** after batch effect adjustment with ComBat algorithm.

In an effort to increase the sample size while reducing the loss of data due to variability of probe sets in different microarray platforms, we selected and compiled three microarray datasets with a total of 50 samples from late onset AD hippocampal tissue (GSE28146, GSE5281, GSE48350) (Liang et al., 2008; Blalock et al., 2011; Berchtold et al., 2013) and three microarray datasets with a total of 87 hippocampal samples (GSE110298, GSE5281, GSE48350) (Liang et al., 2008; Berchtold et al., 2013; Berchtold et al., 2019) from non-demented individuals for the control (NDC) coexpression network (Table 1, Supplementary Table S1). These were the datasets that were generated using the same microarray platform with largest total sample size from all available sets. For the TLE gene coexpression network, we acquired a publicly available microarray dataset of 129 samples from hippocampus of patients who had been diagnosed with TLE and had undergone epilepsy resective surgery (GSE63808) (Johnson et al., 2015). The datasets used in this study are listed in Table 1, with a detailed list of sample types/IDs in Supplementary Table S1. All expression sets were acquired from NCBI Gene Expression Omnibus via GEOquery R package (version 2.52.0) and the probe annotations were mapped to Entrez IDs (Davis and Meltzer, 2007). Initial data visualization was facilitated by NetworkAnalyst web tool (Zhou et al., 2019). In the instances where multiple probes were mapped to the same gene, the average of multiple probe intensities was used to perform gene-level summarization. The expression sets were then filtered for low abundancy genes (the fifth percentile of all annotated genes with lowest relative abundance), log2 transformed and VSN normalized. The ComBat algorithm within SVA R package (version 3.32.1) was employed for batch effect correction (Figures 1A,B), followed by Principal component analysis for visualization (Leek et al., 2012). An Empirical Bayes-moderated linear regression function (empiricalBayesLM) within WGCNA R package (version 1.67) was used for covariate (age and sex) adjustment (Langfelder and Horvath, 2008). The resultant filtered, normalized and covariate-adjusted matrices with a total of 17,821 matched probes/genes were used to generate coexpression networks for the three conditions.

**TABLE 1:** List of the datasets used for this analysis. All raw microarray datasets were downloaded from NCBI Gene Expression Omnibus using the GEOquery R package.

Accession number	Publication	Sample type	Condition	Number of samples	Coexpression network
GSE63808	Johnson et al. (2015)	*Hippocampus* biopsy	Temporal lobe epilepsy	129	TLE
GSE28146	Blalock et al. (2011)	*Hippocampus*, laser capture CA1 neurons	Alzheimer’s disease	21	AD
GSE110298	Berchtold et al. (2019)	*Hippocampus*, homogenate	control	34	NDC
GSE5281	Liang et al. (2008)	*Hippocampus*, laser capture CA1 neurons	Alzheimer’s disease	10	AD
			control	13	NDC
GSE48350	Berchtold et al. (2013)	*Hippocampus*, homogenate	Alzheimer’s disease	19	AD
			control	40	NDC

### Weighted Gene Coexpression Network Analysis (WGCNA)

Three weighted gene coexpression networks were constructed, one for each condition: temporal lobe epilepsy (TLE), Alzheimer’s Disease (AD) and non-demented controls (NDC). First, a sample dendrogram was created for each condition via hierarchical clustering of all samples in order to identify and remove outliers (Supplementary Figures S1C, S2C, S3C). A correlation matrix was constructed based on pair-wise Pearson correlation coefficients of the expression level of all genes across all samples in the set, reflecting the coexpression similarity measure between all pairs of genes. A series of soft thresholding powers were then used to determine the optimal power at which the correlation matrices fit the scale-free topology model, i.e., when the characteristics of the network become independent of its size. The correlation matrix reached a 90% fit to scale-free topology at ß = 5 for AD and NDC datasets, and ß = 7 for TLE dataset (Supplementary Figures S1A,B, S2A,B, 3A,B). An adjacency matrix was then built for each condition by raising the correlation coefficients to the determined soft power of ß = 5 for AD and NDC, and ß = 7 for TLE. A correlation network representing each condition was constructed based on the respective adjacency matrix, where each node corresponds to a single gene, and the edges are determined by the adjacency value between each pair of nodes, reflecting the connection strength and distance between them. All three networks were constructed as “signed” and clustered into modules of coexpressed genes through average linkage hierarchical clustering via “dynamic tree cut” algorithm (Langfelder et al., 2008). The minimum module size was set to 30 genes. The modules were then functionally annotated through pathway enrichment analysis. Central hubs were determined for each module by identifying the top 10 member genes with highest intramodular connectivity and significant correlation to module eigengene—the first principal component of the expression matrix of the corresponding module. The final networks were visualized in Cytoscape software version 3.6.9 (Shannon et al., 2003).

Given that the AD and NDC coexpression networks were generated by combining data from multiple studies, in order to capture any dataset-specific effects on the generation of specific modules, the Pearson correlation was calculated between each dataset and module in the given coexpression network.

### Module Preservation Analysis

In order to understand the extent of preservation of TLE modules in the AD and NDC networks, we used two approaches. The first approach was cross-tabulation, which is a simpler method that creates a contingency table reporting the number of overlapping genes from each TLE module with each of the AD or NDC module. The significance of the overlap is calculated through Fisher’s exact test. The second, more complex approach utilizes network separability, density and connectivity-based preservation statistics available within the modulePreservation function in the WGCNA R package (version 1.67) introduced by Langfelder et al. (2011) and described in detail in Oldham et al. (2006). This method requires adjacency matrices of both reference (TLE) and test networks (AD and NDC) as input, however module assignment is only necessary for the reference-TLE network (Langfelder et al., 2011). The observed preservation value for each module detected in the TLE network was calculated in the AD and NDC networks. We then employed a permutation test (number of permutations = 500) which randomly permutes the module assignment in the AD and NDC networks to assess if the observed value of preservation statistic is higher than what is expected by chance and assigns a permutation test *p*-value. The observed preservation values were then standardized with regard to the mean and variance and a significance Z score was defined for each preservation statistic (Supplementary Tables S5, S6). The overall significance of the observed statistics was assessed by combining multiple preservation Z statistics into a single overall measure of preservation defined as Z_summary_. Thus, each module identified in the TLE network has a pair of Z_summary_ scores which describe the preservation of the given module in the AD $\left(Z_{\mathrm{summary}}^{\mathrm{AD}}\right)$ and NDC $\left(Z_{\mathrm{summary}}^{\mathrm{NDC}}\right)\;$ networks, respectively. Modules with Z_summary_>10 are considered well-preserved (Langfelder et al., 2011), thus the higher the Z_summary_ score, the stronger the evidence of preservation of the given TLE module in the two test networks. In order to compare the degree of preservation of each TLE module between the AD and NDC networks, we introduce a surrogate quantifier of “differential module preservation”—***ΔZ***_***summary***_, which is the arithmetic difference between the two preservation scores:

$$\Delta Z_{summary}=Z_{summary}^{AD}-Z_{summary}^{NDC}$$

Thus, the modules with a positive ***ΔZ***_***summary***_ value are more preserved (show Gain Of Preservation, GOP) and the modules with a negative ***ΔZ***_***summary***_ value are less preserved (show Loss Of Preservation, LOP) in the AD network compared to the control-NDC network.

### Functional Annotation and Enrichment Analysis

In order to determine the functional significance of the detected modules, a pathway enrichment analysis of the genes constituting each module was performed using the g:Profiler web tool (Reimand et al., 2016). Since the Gene Ontology database has a hierarchical order, in the instances of a large number (hundreds) of enriched pathways, we clustered the GO terms lower in hierarchy into their representative “parent” terms higher in the hierarchy using a similarity threshold of 90% (Supek et al., 2011).

## Results

### Temporal Lobe Epilepsy Coexpression Network

**FIGURE 2:**
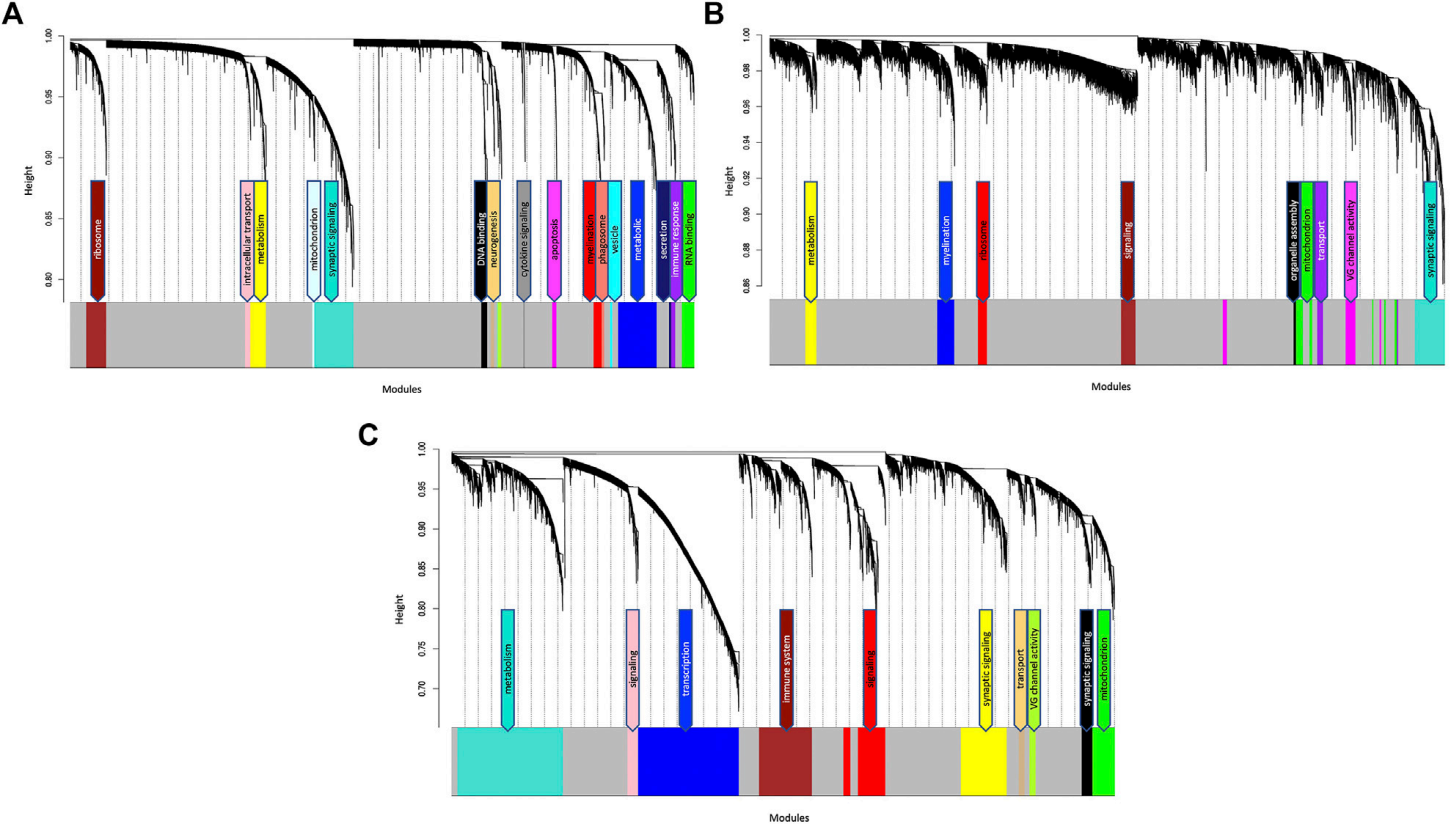
Hierarchical cluster dendrogram of **(A)** TLE **(B)** AD and **(C)** NDC gene coexpression networks. Each black branch (vertical line) corresponds to one gene. The colour rows below the dendrogram indicate module membership. The modules are functionally annotated and named based on the enriched GO/KEGG pathways of the member genes. The grey module contains genes that have no specific module assignment i.e., the expression pattern of these genes does not show sufficient variability.

After filtering, probe annotation and removal of outliers, a total of 127 samples were included in the TLE coexpression network. Hierarchical clustering identified 18 modules of highly coexpressed genes, ranging from 27 to 999 nodes in size (Figure 2A; Supplementary Table S2). The modules were annotated based on the functional enrichment analysis of member genes, with the most enriched pathways in the top hierarchy levels being used as functional labels. Functional annotation of member genes from all modules rendered significantly enriched Gene Ontology terms and KEGG pathways, ranging from a few dozen to several hundreds of pathways (Supplementary Table S2). Interestingly, the largest module (Turquoise, 999 nodes) which is enriched for genes involved in synaptic signalling and neurotransmission processes in the GO biological process category, also identified “Pathways of neurodegeneration” and “Alzheimer Disease” as some of the most significantly enriched KEGG pathways (FDR = 1.04 × 10^−8^, FDR = 6.15 × 10^−7^, Supplementary Table S2). This finding suggests that in TLE, the genes responsible for neurotransmission and signal transduction behave in a manner characteristic to Alzheimer’s Disease pathology. A smaller module (Tan, 84 nodes) which is enriched for neurogenesis and GABAergic signalling pathways was identified as enriched for 35 terms from the human phenotype ontology database, all indicating seizure or epilepsy-related ontologies (Supplementary Table S2).

### Alzheimer’s Disease Coexpression Network

After filtering and removal of outliers, a total of 49 samples were included in the AD coexpression network. WGCNA identified 10 modules of highly coexpressed genes, ranging from 32 to 708 nodes in size (Figure 2B; Supplementary Table S3). The largest module (also labelled as Turquoise) is enriched for neurotransmission and synaptic signalling-related processes such as “chemical synaptic transmission” (FDR = 1.1 × 10^−34^), “voltage gated channel activity” (FDR = 4.25 × 10^−8^), “ion transmembrane transport” (FDR = 1.02 × 10^−21^) and “neurotransmitter transport” (FDR = 2.18 × 10^−14^, Supplementary Table S3). No significant correlation (P_adj_ < 0.05) between specific datasets and modules were detected (Supplementary Figure S4A).

### Non-Demented Control Coexpression Network

After filtering and removal of outliers, a total of 84 samples were included in the NDC coexpression network. WGCNA identified 11 modules of highly coexpressed genes which were substantially larger in size (ranging from 160 to 2,836 nodes) than those identified in the TLE and AD coexpression networks. The two largest modules (Blue and Turquoise) were enriched for processes involved in gene expression and metabolism, respectively. Three smaller modules (Yellow, Black and Greenyellow) were functionally enriched for synaptic signalling processes and voltage gated channel activity (Figure 2C; Supplementary Table S4). Similar to what was observed in the AD network, no significant correlation (P_adj_ < .05) between specific datasets and modules were detected in the NDC network (Supplementary Figure S4B).

### TLE Modules are Preserved to Various Degrees in AD and NDC Networks

**FIGURE 3:**
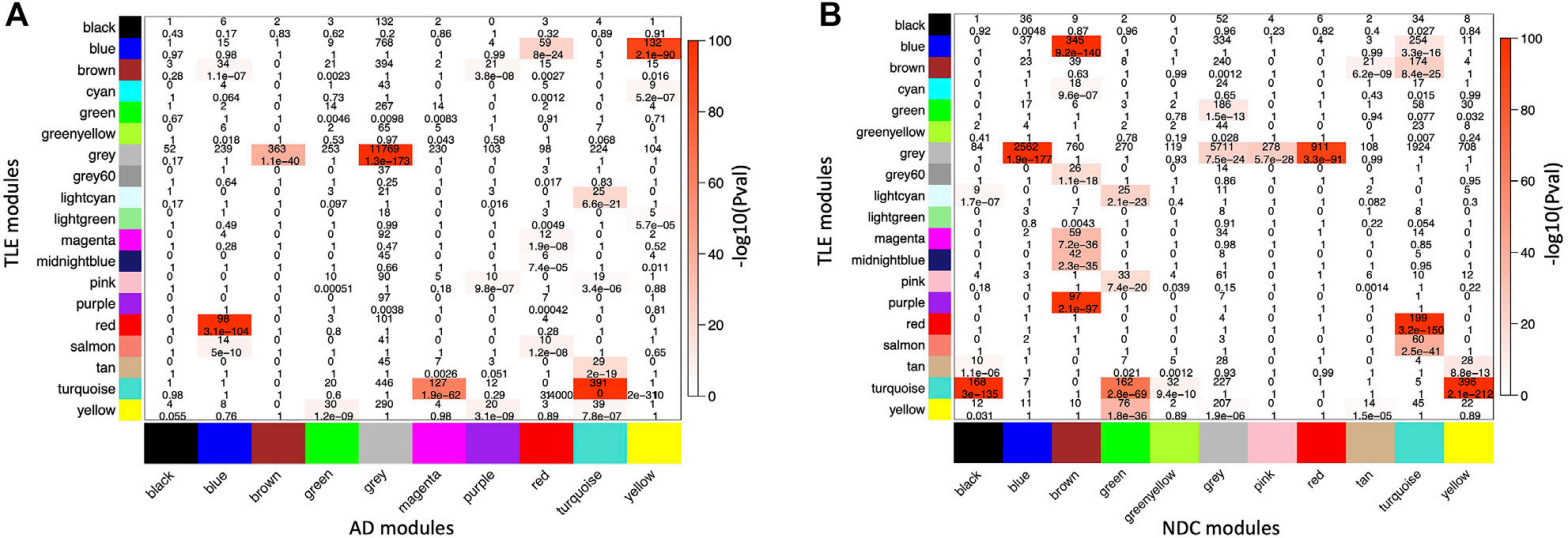
Cross-tabulation of TLE modules (in *Y* axis) against **(A)** AD and **(B)** NDC modules (in *X* axis). Each axis is labelled by the corresponding module colour. Each block in the table shows the number of overlapping genes in the intersection of corresponding **(A)** TLE and AD and **(B)** TLE and NDC modules. The table is colour-coded with -log10 of the *p* value associated with the Fisher exact test.

**FIGURE 4:**
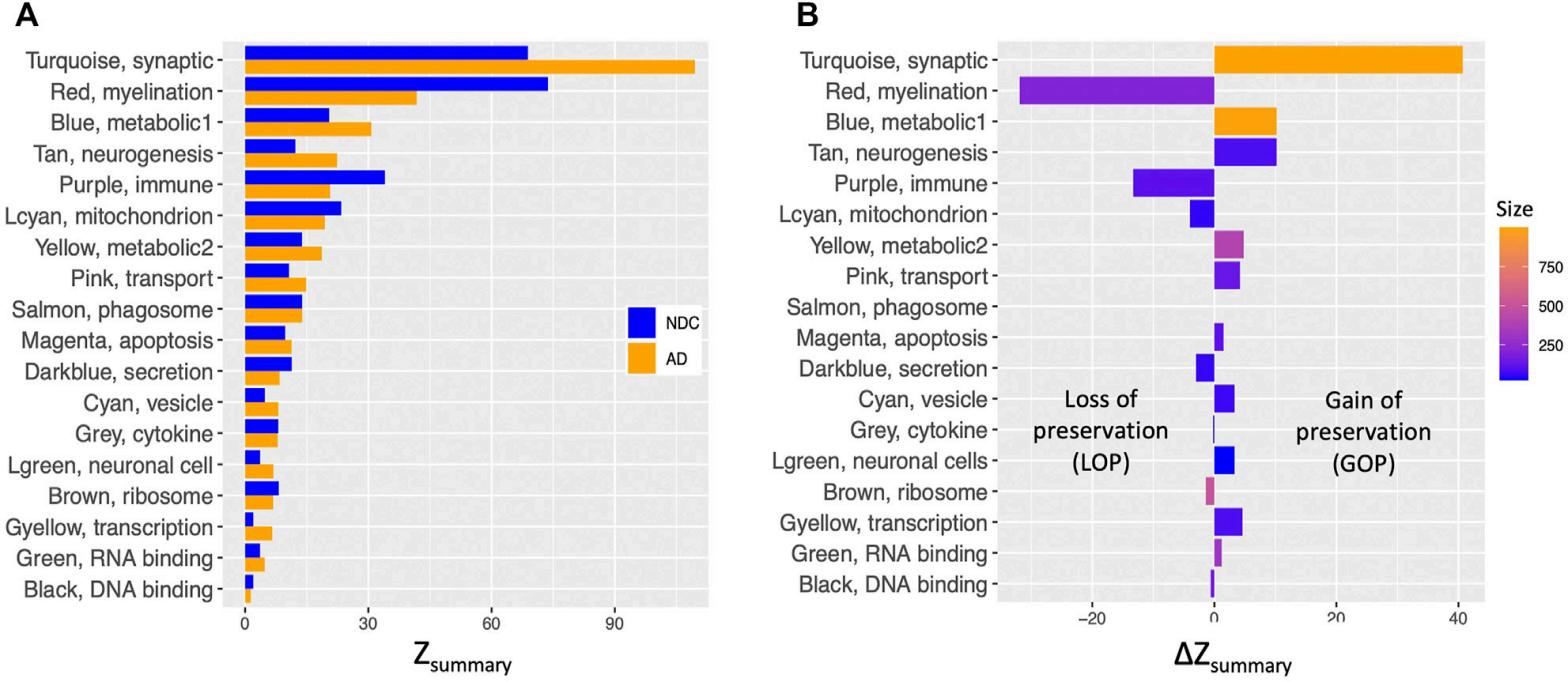
Comparison of density and connectivity-based preservation of TLE modules in AD and NDC networks. **(A)** The overall significance of the observed preservation statistics was assessed for each TLE module (*Y* axis) by combining density and connectivity-based preservation Z statistics into a single overall measure of preservation defined as Z_summary_ shown in pairs on *X* axis for NDC (blue bars) and AD (orange bars) networks. **(B)** The arithmetic difference of preservation Z_summary_ values, ΔZ_summary_ in *X* axis between NDC and AD networks for each of the TLE modules in *Y* axis. Positive ΔZ_summary_ indicates gain of preservation (GOP, more preserved) and negative ΔZ_summary_ indicates loss of preservation (LOP, less preserved) of the given TLE module in the AD network compared to NDC network.

In order to capture any similarities within the module composition and network architecture of the two pathological gene networks, while allowing for discrimination between unperturbed (homeostatic) and perturbed (dysregulated) modules, two pairs of comparisons were made [TLE vs. AD] and [TLE vs. NDC]. Several large modules showed significant overlap in member genes between both pairs of networks as measured by cross-tabulation of every module from TLE network with those from AD (Figure 3A) and NDC (Figure 3B) networks. The most significant (P ≈ 0) overlap was observed between the two Turquoise modules in the TLE and AD networks, both of which are enriched for synaptic signalling pathways (Figure 3A). The density and connectivity-based preservation scores as calculated by the modulePreservation function are shown in Figure 4A for each of the TLE modules. The arithmetic difference between the two preservation scores titled ΔZ_summary_ was used to define modules which show Gain Of Preservation or Loss Of Preservation in the AD network when compared to the NDC network (Figure 4B). In the following sections, we characterise the overlap and preservation of TLE modules in AD and NDC networks based on the biological pathways they represent.

#### Synaptic Signalling

The largest GOP (by ∼60%, ΔZ_summary_ = 40.7) was observed in the Turquoise module, which functionally annotates to synaptic signalling processes. With a Z_summary_ of 109.5, this module is extremely well preserved between TLE and AD coexpression networks (Figure 3A). The corresponding preservation score for this module between TLE and NDC coexpression networks is Z_summary_ = 68.8. With voltage-gated sodium channel 3 subunit B (*SCN3B*) as a hub gene, this module is enriched for voltage-gated ion channel activity, synaptic signalling and neurotransmission pathways. There is significant (*p* = 2 × 10^−310^) overlap between the genes constituting the TLE Turquoise module with the corresponding neurotransmission-associated module in the AD network (also labelled Turquoise, Figure 3A). In the NDC network, the TLE Turquoise module appears to split into three distinct modules (Figure 3B), two of which (Black and Yellow) functionally annotate to synaptic signalling pathways, while the third, Green module is enriched for mitochondrial-based processes (Figure 2C). These results suggest homologous restructuring and perhaps perturbation of synaptic signalling pathways in TLE and AD-affected hippocampus compared to NDC.

The WGCNA clustering algorithm identified another smaller module (Tan) in the TLE coexpression network, which functionally annotates to synaptic signalling and neurogenesis-related pathways. The Tan module shows GOP (ΔZ_summary_ = 10.2) and contains a GABA receptor subunit (*GABRB3*) and an alpha subunit of VGSC (*SCN2A*) as it’s top regulatory hub genes. Although this module is enriched for GO terms that are similar to Turquoise module, according to the gene correlation dendrogram (Figure 2A), these modules branch off from each other at a high hierarchy level, and are therefore two distinct, independent modules. There is similar overlap of genes between the TLE Tan module with corresponding neurotransmission-associated modules in both AD and NDC networks (Figures 3A,B).

#### Metabolism

There are two distinct TLE modules (Blue and Yellow) with member genes that are enriched for metabolic processes (Figure 2A), both of which show GOP (ΔZ_summary_ = 10.2 for Blue and ΔZ_summary_ = 4.8 for Yellow, Figure 4B). The larger, Blue module has 989 member genes, with thyroid hormone receptor interactor 6 (*TRIP6*) as its top regulatory hub gene and functionally annotates to metabolic, cellular organization, transport, and other homeostatic processes. The smaller, Yellow module functionally annotates to more specific mitochondrial functions such as “oxidative phosphorylation,” “ATP synthesis coupled proton transport” and “oxidoreductase activity” as well as multiple mitochondrion organization pathways. The hub gene for this module is Acid phosphatase 1 (*ACP1*), the main function of which is hydrolysis of tyrosine and its phosphates. When cross referencing the KEGG database, the genes comprising the Yellow module rendered “Alzheimer’s Disease” as one of the most enriched KEGG pathways (P_adj_ = 8.54 × 10^−6^, Supplementary Table S2).

Another small metabolic TLE module (Lightcyan) with 53 member genes shows LOP (ΔZ_summary_ = −4.0, Figure 4B) between AD and NDC networks. The Lightcyan module is highly enriched for mitochondrial-based processes and has *YWHAG* as a central hub gene. *YWHAG* codes for the Tyrosine 3-Monooxygenase activation protein Gamma, also known as Protein Phosphatase 1, regulatory subunit 170, which belongs to a family of proteins that mediate signal transduction and are involved in many vital cellular processes such as metabolism, apoptosis and cell cycle regulation. The top enriched pathways of this module include “TCA cycle and respiratory electron transport,” “mitochondrial ATP synthesis coupled electron transport,” “ATP metabolic process” and other oxidative phosphorylation-related pathways.

#### Myelination

Another module of interest showing substantial LOP is the Red module, with 206 member genes and ΔZ_summary_ of −31.9 (Figure 4B). This module is enriched for genes involved in myelination and axon ensheathment pathways, and as a central hub gene has NK6 homeobox 2 (*NKX6-2*), which is a transcription factor involved in axon-glial interactions at nodes of Ranvier. The Red module shows significant overlap of member genes with the corresponding myelination-associated module in the AD coexpression network (labelled Blue, Figures 3A) and a large, metabolic module (labelled Turquoise, Figure 3B) in the NDC coexpression network.

#### Immune System

The final TLE module of interest showing LOP (ΔZ_summary_ = −13.3) is the immune system-associated Purple module, with 107 member genes and transmembrane immune signalling adaptor (*TYROBP*) as a central regulatory hub gene. This module is enriched for immune system processes such as leukocyte activation, cytokine production and signalling, antigen processing and presentation, interferon signalling and other inflammatory pathways. With 97 out of 107 genes in common, there is significant overlap (*p* = 2.1 × 10^−97^) between the genes constituting the Purple module in the TLE network and the Brown immune system-associated module in the NDC network (Figures 2C, 3B). The same 97 genes from TLE Purple module overlap with the unassigned (Grey) module in the AD network (Figure 3A), indicating that immune signalling involving these genes may be dysregulated in AD.

## Discussion

Epilepsy and Alzheimer’s disease often co-occur and are thought to have a bi-directional relationship, however, there are significant gaps in knowledge regarding the pathophysiological mechanisms and cause-effect relationships between the two conditions. This is due to the lack of comprehensive population-based studies (Subota et al., 2017) as well as scarcity (or absence) of clinical and electroencephalographic data accompanying the gene expression profiling datasets.

The aim of this study was to characterize the shared molecular signature of AD and TLE and identify commonly dysregulated pathology-specific gene modules which could explain the correlated incidence of the two diseases.

Given that the electrophysiological and morphological symptoms common to TLE and AD severely impact the hippocampus, we selected publicly available transcriptomic datasets from hippocampal tissue of relevant pathological and control states. In order to generate a reliable and robust gene coexpression signature of a disease, it is imperative to have datasets with a large number of samples. At the time of our analysis, there was no single publicly available transcriptomic dataset profiling the hippocampus of AD patients that had more than 25 samples for each group, therefore, we employed a common strategy to combine multiple datasets together. While increasing statistical power, combining samples from different datasets usually introduces artificial variance, due to the differences in microarray platforms and sample types. In order to correct for these artefact differences between the AD and NDC groups, we undertook several strategies: first, we selected datasets that were generated using the same microarray platform (GPL570 [HG-U133]). Second, where possible, we included AD and control samples from the same dataset (GSE5281 and GSE48350), to account for variation introduced by sample type differences (laser-capture micro dissected neurons vs. homogenate). Lastly, we used robust statistical methodology to adjust the expression sets for batch effects and covariates (Figure 1, detailed in *Methods*).

According to our findings, AD and TLE show similar rewiring of synaptic transmission and metabolism-related gene networks. Perturbed synaptic transmission is not surprising, considering that circuit dysfunction and hyperexcitability are common features of both pathologies. The *SCN3B* gene which codes for a beta subunit of voltage gated sodium channel NaV1.1 was identified as the main regulatory hub gene of the synaptic transmission-associated Turquoise module identified in the TLE network, which shows the highest preservation score and largest GOP in AD coexpression network. As the most connected node of the Turquoise module, *SCN3B* is an interesting regulatory hub candidate. The beta subunits of voltage gated (VG) ion channels carry a multitude of essential functions. In addition to being VG channel modulators, the beta subunits also function as cell adhesion molecules and regulators for voltage-gated sodium channel (VGSC) alpha subunit gene expression, due to being substrates for sequential cleavage by beta secretase (BACE1) (Wong et al., 2005). The expression of BACE1 is reportedly increased in AD pathology, resulting in abnormal VGSC beta subunit cleavage, which has been shown to result in reduced levels of functional Nav1.1 channels on the surface of GABAergic interneurons, leading to network disinhibition and higher susceptibility to seizures in mouse models of AD (Kim et al., 2007; Kim et al., 2011; Corbett et al., 2013).

Mutations in the genes that code for both alpha and beta subunits of VGSC are implicated in various neuropathologies (O’Malley and Isom, 2015). Pathological levels (both increase and decrease) of *SCN3B* mRNA have been implicated in multiple neurodegenerative diseases such as amyotrophic lateral sclerosis and Alzheimer’s disease as well as cancer (Adachi et al., 2004; Dunckley et al., 2006; Nutini et al., 2011). Another central hub gene of this module, Ephrin type A receptor 4 (*EPHA4*), belongs to the protein-tyrosine kinase family and has been implicated in various synaptic dysfunction-related pathologies, including AD and TLE (Fu et al., 2014; Shu et al., 2016). Under homeostatic conditions, the EphA4 acts as a negative regulator of neurotransmission and synaptic plasticity in the hippocampus (Murai and Pasquale, 2011). It has been shown to become deregulated and overactivated, resulting in synaptic failure, in mouse models of AD (Fu et al., 2014) and TLE (Shu et al., 2016) pathology. In these models, blockade/knockdown of the receptor rescues the synaptic impairment. It is therefore conceivable that deregulation and downstream signalling of EphA4 is one of the shared pathological mechanisms between AD and TLE, which leads to abnormal neurogenesis and impairments in synaptic signalling.

The second notable module of interest is the neurogenesis-associated Tan module. Despite the small size of this module, the expression pattern of its 84 member genes is closely correlated in TLE and AD networks, resulting in formation of an independent module that is highly preserved and shows substantial GOP in the AD network compared to NDC. The Tan module also appears to be a seizure-associated module. Central hub genes among the top 10 most connected nodes centrally located in this module include *GABRB3* (gamma-aminobutyric acid type A receptor subunit beta3), *SCN2A* (sodium voltage-gated channel alpha subunit 2), *PRICKLE1* (Prickle planar cell polarity protein 1) and *FRMPD4* (FERM and PDZ domain containing 4). These genes have been shown to be associated with various types of seizures and EEG abnormalities (Corbett et al., 2010; Polan et al., 2014; EuroEPINOMICS-RES Consortium, 2014). Notably, all 35 significantly enriched (P_adj_ < .05) phenotypes associated with the Tan module from human phenotype ontology database implicate EEG abnormalities and epileptiform activity (Supplementary Table S2). These phenotypes have been observed in patients with AD and various epilepsy disorders (Vossel et al., 2012; Vossel et al., 2013; Vossel et al., 2016) and reported in animal models of AD (Ziyatdinova et al., 2011; Bezzina et al., 2015; Kam et al., 2016; Ziyatdinova et al., 2016; Drinkenburg et al., 2017). Epileptiform activity is one of the major clinical features shared between AD and TLE, and is thought to contribute to more rapid disease progression and cognitive decline (Volicer et al., 1995; Vossel et al., 2013; Vossel et al., 2016), therefore, further investigation of this distinct disease module has the potential of facilitating more targeted treatment options. Specifically, large scale transcriptomic studies which include detailed clinical and electroencephalographic (EEG) data from the patients who donated the brain tissue would be instrumental in assessing the correlation between the neurogenesis-related gene module and specific abnormalities seen in the EEG.

The second largest difference in TLE module preservation scores was observed for the Red module (ΔZ_summary_ = −31.9), which annotates to myelination and axon ensheathment processes. While a Z_summary_ score of 41.8 is still indicative of very strong preservation (Langfelder et al., 2011) in the AD network, the large difference in the preservation score of the corresponding module in the NDC network (Z_summary_ = 73.7) suggests that the operation of myelination and axon ensheathment processes is at least partially altered in the setting of AD compared to TLE and NDC. Indeed, it has been reported before that myelination-associated gene modules are severely perturbed in the prefrontal cortex of AD (Zhang et al., 2013). Impairment in cholesterol metabolism which is detected in AD brain (Martins et al., 2009; Sodero et al., 2012) could lead to alterations in the myelination processes, since 25% of the total cholesterol in the human body is allocated to the brain, where it is mostly localized to the plasma membranes of neurons and glia, and myelin sheaths covering the axons (Björkhem and Meaney, 2004). This may explain why the myelination module from TLE coexpression network is less preserved in the AD network compared to NDC.

Given the evidence of strong neuroinflammatory presence in both AD and TLE pathologies (Akiyama et al., 2000; Glass et al., 2010; Maroso et al., 2010; Maroso et al., 2011; Vezzani et al., 2011), it is somewhat surprising that the Purple immune system-associated module is substantially less preserved between TLE and AD, compared to TLE and NDC networks. The central regulatory hub gene of the Purple module is *TYROBP*, which has been previously identified as a causal regulatory hub within a microglia-associated module generated from prefrontal cortex samples from late onset AD patients (Zhang et al., 2013). Several studies investigating gene regulatory networks of AD showed disruption of normal microglial gene modules (Zhang et al., 2013; Efthymiou and Goate, 2017; Mukherjee et al., 2019). We therefore speculate that in the AD-affected hippocampus, there is rewiring of microglial gene networks that is distinct from TLE and NDC.

When considering the periodic increase in neuronal energy demand to sustain seizures, it is not surprising that metabolic pathways are among the primary characteristics of the molecular signature of TLE. A relatively new but growing hypothesis is that neurodegenerative disorders, including late onset AD and several types of acquired epilepsy arise from metabolic dysfunction and are aggravated by oxidative stress and mitochondrial dysfunction (Mattson, 2004; Lin and Beal, 2006; Dejakaisaya et al., 2021). Indeed, the majority of genes and proteins associated with the term “Alzheimer’s Disease” in various biological pathway databases belong to metabolic pathways (Ogata et al., 1999; Thomas et al., 2003; Harris et al., 2004; Joshi-Tope et al., 2005). A large number of these pathways were significantly enriched (FDR < 0.05) in both metabolic modules (Blue and Yellow) of the TLE network and the Yellow metabolic module in the AD network. This suggests that perturbations in redox balance, oxidative phosphorylation and other mitochondrial processes are important players in epileptogenesis and should be studied further.

The results of our analysis suggest that in addition to clinical and morphological features, Alzheimer’s Disease and temporal lobe epilepsy share specific defects in the molecular mechanisms that regulate excitability, synaptic signalling, neurogenesis and mitochondrial pathways. Perturbed metabolism and mitochondrial dysfunction may contribute to impairment in neurotransmission and consequently lead to the electrophysiological abnormalities and cognitive symptoms seen in both AD and epilepsy disorders. As the pathology progresses, accumulation of reactive oxygen species could increase epilepsy sensitivity and result in seizure development. On the other hand, epileptic activity may aggravate the cognitive decline and neurodegeneration in AD, thus putting the patients in a vicious cycle of debilitating symptoms and fast progression of disease. In the absence of a clear causal mechanism such as traumatic brain injury or genetic mutation, it is challenging to determine which factor is the metaphorical “chicken” and which is the “egg” due to the enormous variation in immune and metabolic profiles, vast variety of lifestyles, environmental circumstances and combinations of life events. The difficulties of recognizing ictal events (the most common type of seizures experienced by AD patients are nonconvulsive or complex-partial seizures, which are easily missed) and differentiating between seizure-related and dementia-related symptoms present further challenges to our understanding of the mechanisms driving the pathology in these conditions (Vossel et al., 2013; Miranda and Brucki, 2014). As shown here, a systems level network-based approach wherein gene modules represent emergent global properties of biological function, provides a useful tool for hypothesis generation, which then would be subject to experimental validation. We demonstrate that gene coexpression network analysis and network preservation statistics methods can be used for a holistic, hypothesis-free, systems-level examination and comparison of two pathological states, solely based on the gene coexpression network architecture of each state. Additionally, we present a list of central hub genes that have the potential of influencing the larger molecular network they regulate. The identification of pathology-related modules which are associated with the clinical features and phenotypes observed in the context of both diseases and the implication of these modules in the published epilepsy and dementia literature highlights the validity of our approach. However, our findings should be interpreted with two limitations in mind. Phenotypic classification is compromised by a lack of epilepsy-specific information on AD patients and vice versa and tissue-based samples do not allow cell-type specific effects to be identified. Further experimental evidence by means of large-scale transcriptomic profiling of hippocampal tissue from patients with consistent clinical history and electroencephalographic (EEG) data is necessary to establish the identified regulatory hub genes and implicated cellular pathways as causal agents of epileptogenesis.

## Data Availability

Publicly available datasets were analyzed in this study. This data can be found here: https://www.ncbi.nlm.nih.gov/geo/ Accession numbers: GSE63808, GSE5281, GSE28146, GSE48350, GSE110298.

## Supplementary Material

Supplementary Figure S1 The scale free topology fit, mean connectivity and hierarchical clustering tree of all samples in TLE dataset.

Supplementary Figure S2 The scale free topology fit, mean connectivity and hierarchical clustering tree of all samples in AD dataset.

Supplementary Figure S3 The scale free topology fit, mean connectivity and hierarchical clustering tree of all samples in NDC dataset.

Supplementary Figure S4 Heatmap detailing the relationship between the datasets (x axis) and all detected modules (y axis) and in the **(A)** AD and **(B)** NDC networks. Each block in the heatmap shows the direction (red: positive, blue: negative), strength (Pearson correlation coefficient, top number) and FDR-adjusted significance of the Pearson correlation (in brackets) of the given module to each dataset that was used to generate the network.
