# Supplementary figures and images for: Network Preservation Analysis Reveals Dysregulated Synaptic Modules and Regulatory Hubs Shared Between Alzheimer’s Disease and Temporal Lobe Epilepsy
Source: Front Genet. 2022 Mar 2;13:821343. doi: 10.3389/fgene.2022.821343 (PMC8926077; doi:10.3389/fgene.2022.821343)

**A Module-dataset relationships, AD**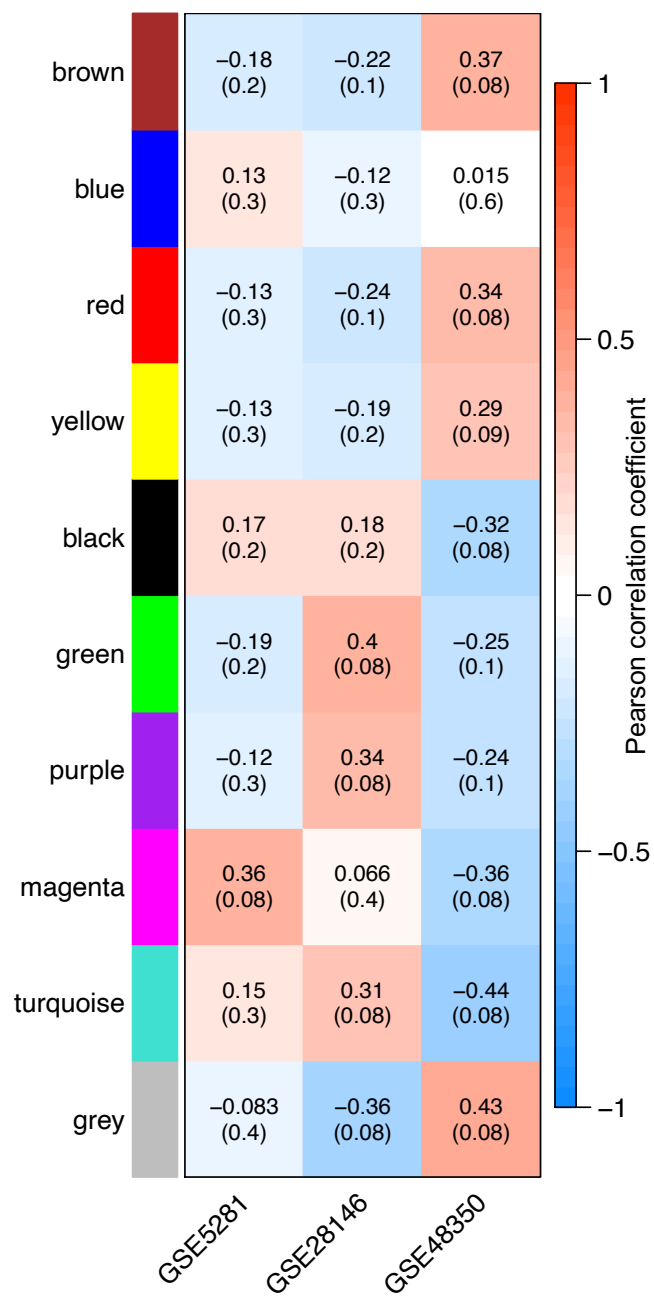**B Module-dataset relationships, NDC**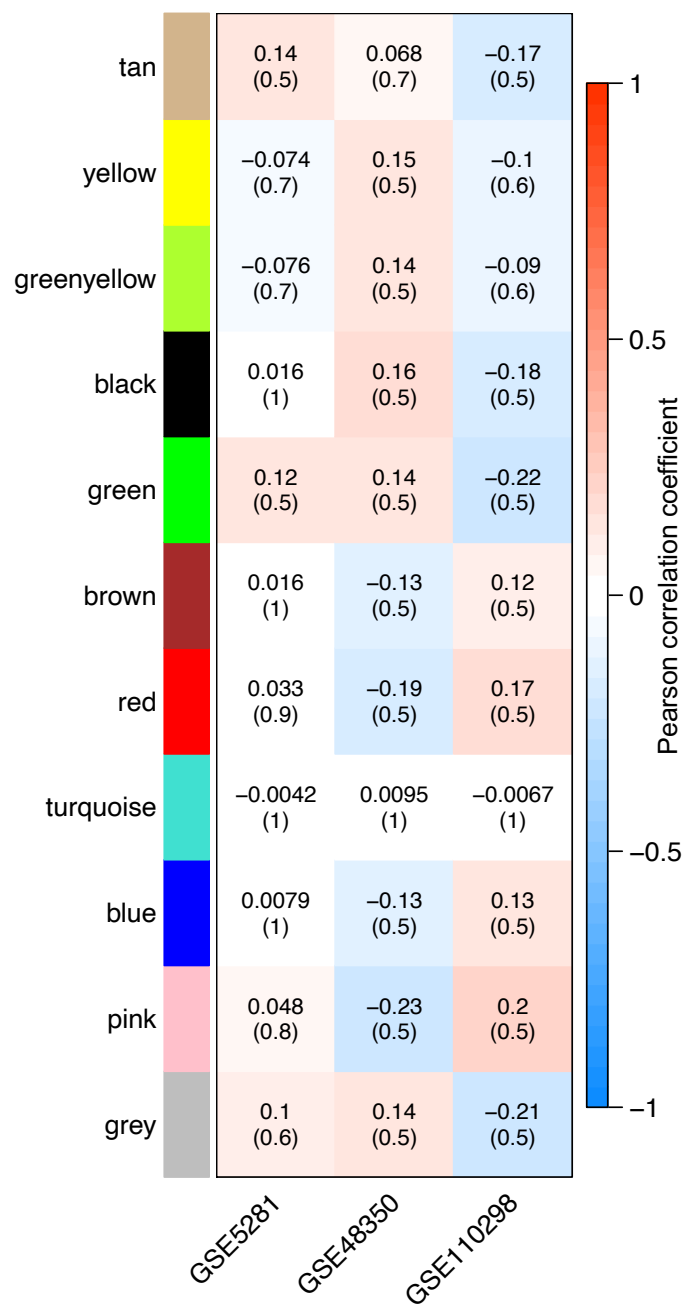

Supplement: Supplementary file 1 [file Image4.pdf]

**A**

### Scale independence AD

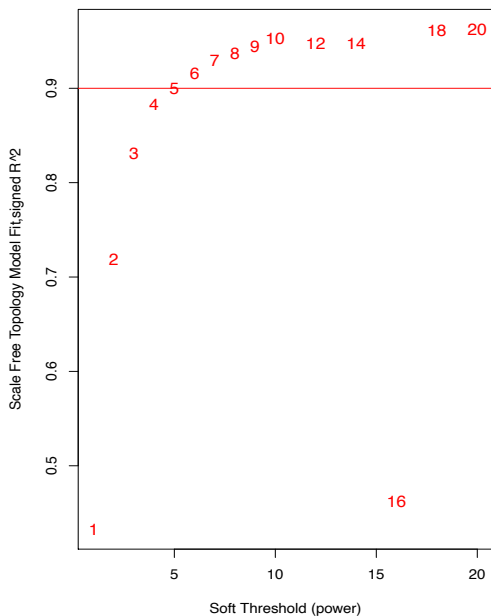

# B

### Mean connectivity AD

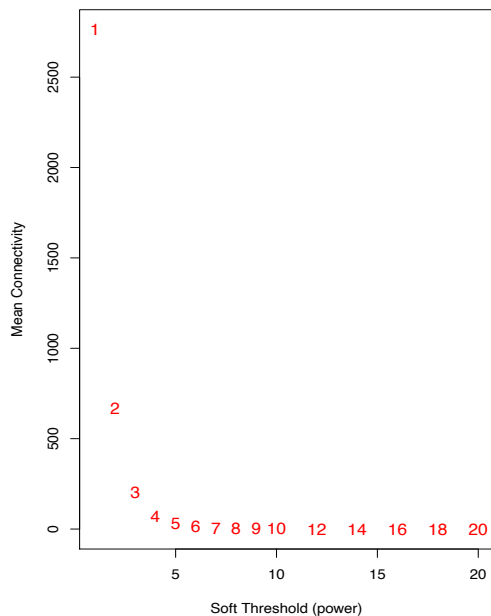

**C**

### Sample clustering to detect outliers

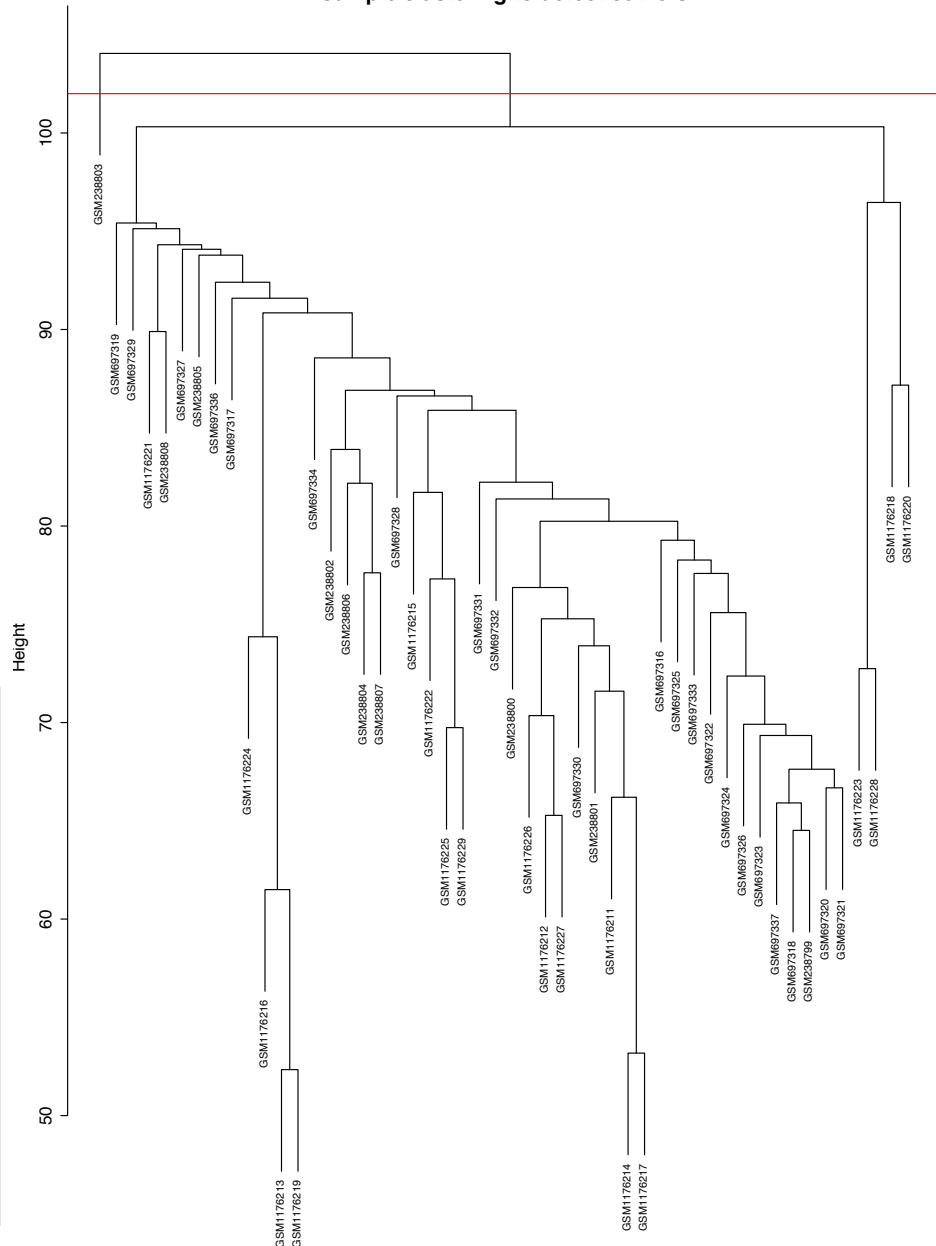

Supplement: Supplementary file 2 [file Image2.pdf]

**A**

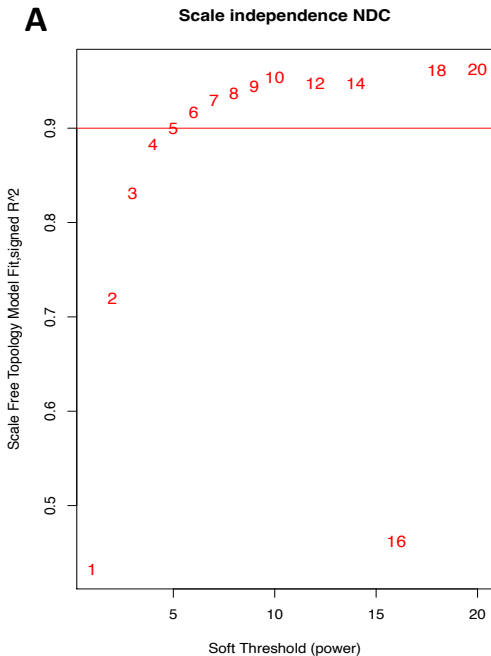

## B

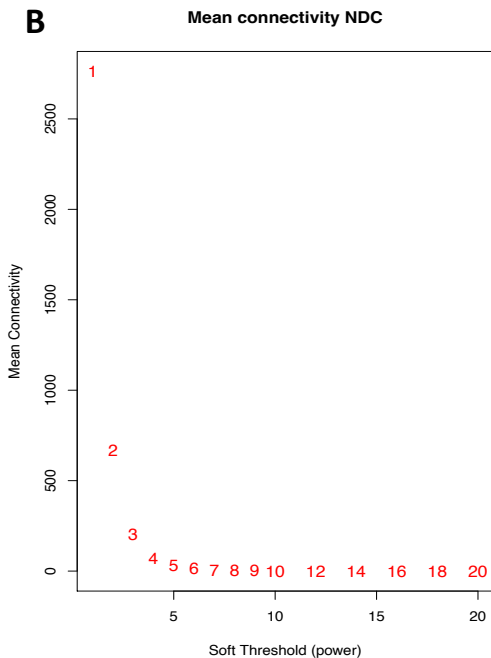

**C**

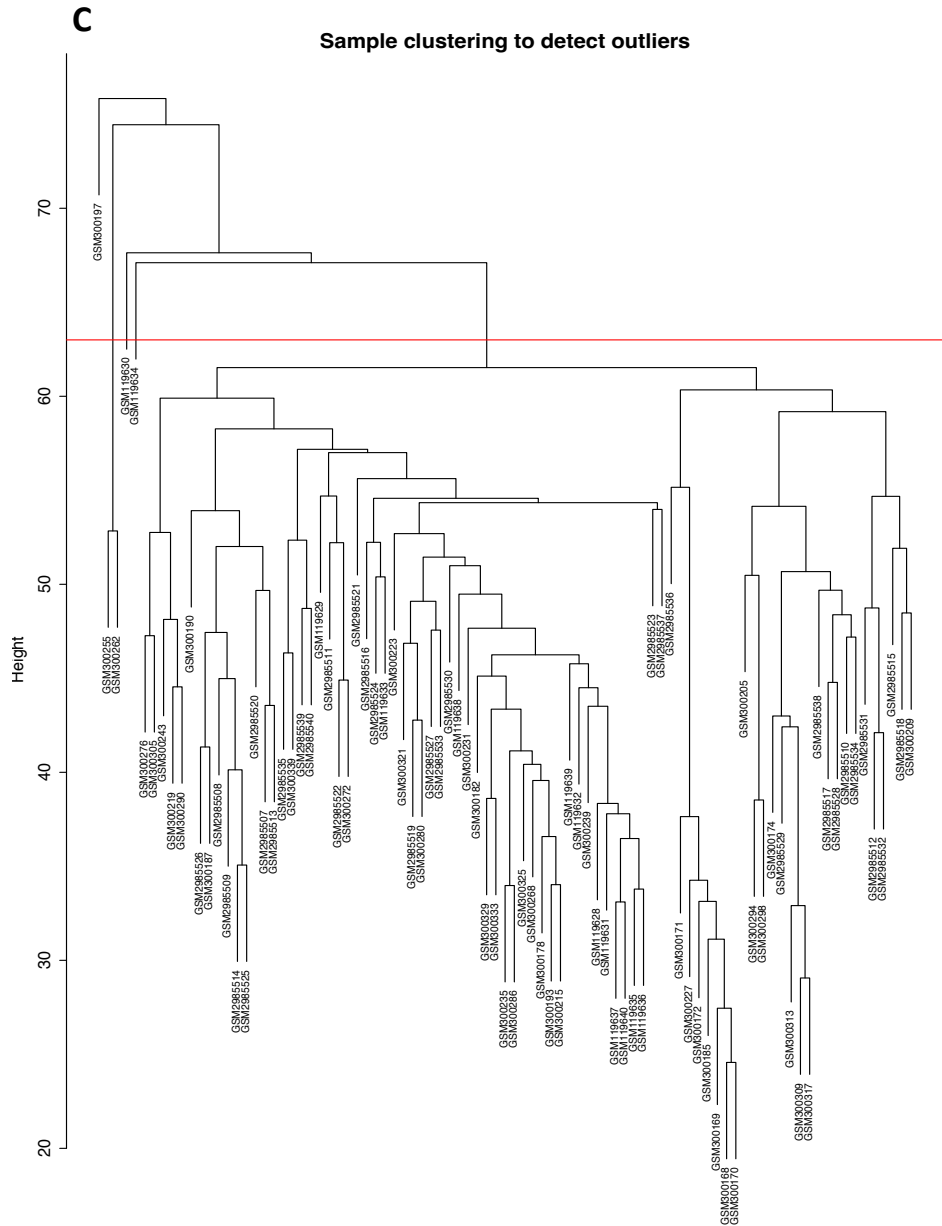

Supplement: Supplementary file 3 [file Image3.pdf]

**A****Scale independence TLE**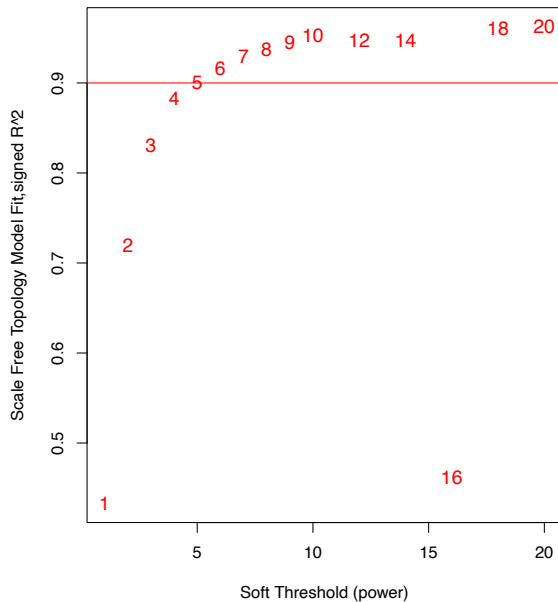**B****Mean connectivity TLE**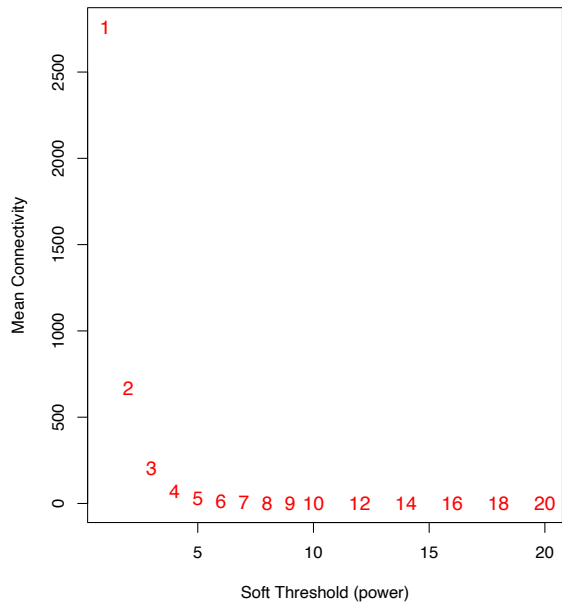**C****Sample clustering to detect outliers**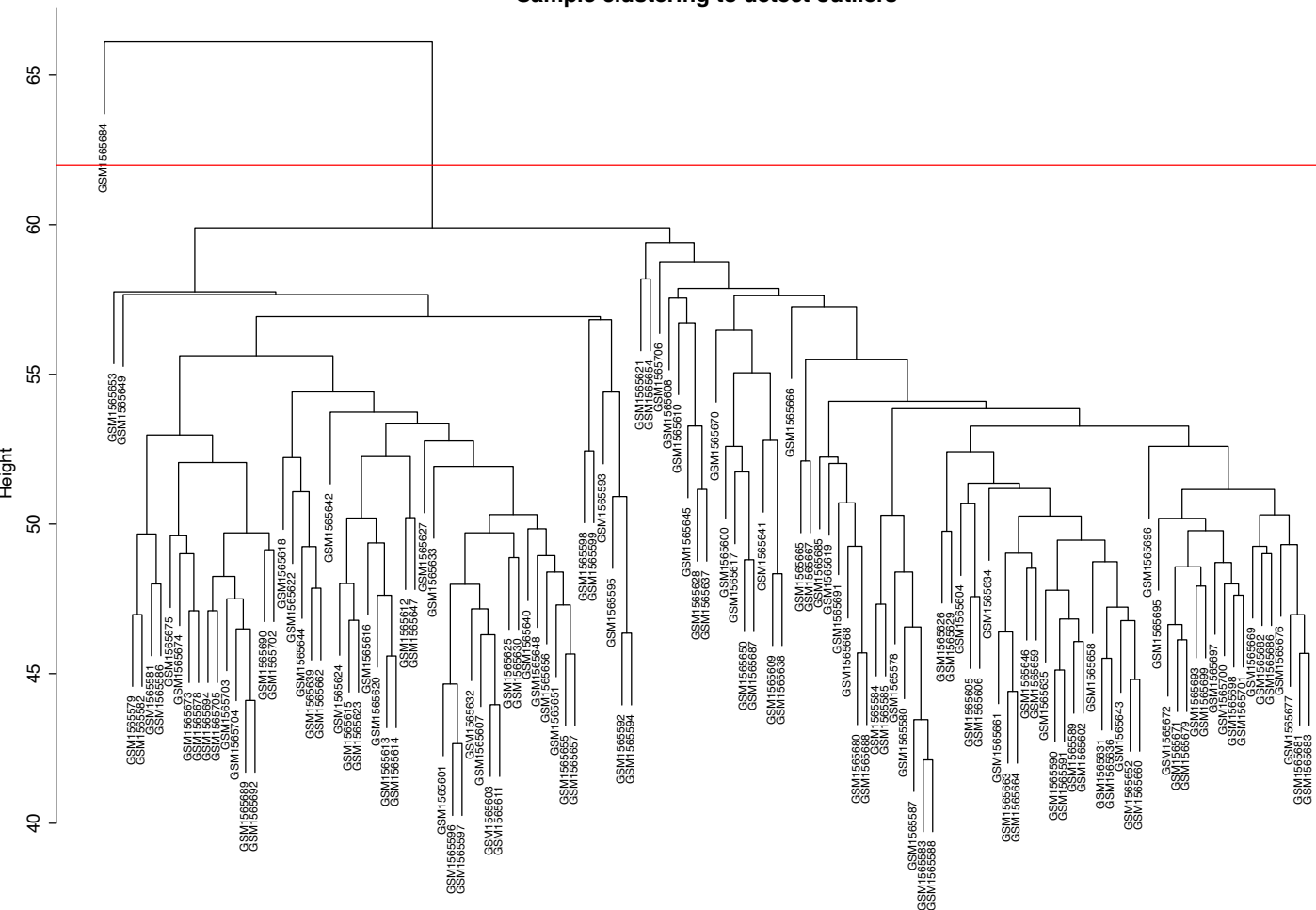

Supplement: Supplementary file 5 [file Image1.pdf]
